# Household Air Pollution, Levels of Micronutrients and Heavy Metals in Cord and Maternal Blood, and Pregnancy Outcomes

**DOI:** 10.3390/ijerph15122891

**Published:** 2018-12-17

**Authors:** Ganiyu Olatunbosun Arinola, Anindita Dutta, Oluwafemi Oluwole, Christopher O. Olopade

**Affiliations:** 1 Department of Chemical Pathology, College of Medicine, University of Ibadan, Ibadan 200284, Nigeria; 2 Department of Medicine and Center for Global Health, University of Chicago, 5841 S. Maryland Avenue, MC 2021 Chicago, IL 60637, USA; anidu14@gmail.com (A.D.); solopade@medicine.bsd.uchicago.edu (C.O.O.); 3 Department of Pediatrics and the Canadian Centre for Health and Safety in Agriculture, College of Medicine, University of Saskatchewan, Saskatoon, SK S7N 2Z4, Canada; olo535@mail.usask.ca

**Keywords:** heavy metals, micronutrients, cord blood, maternal blood, household air pollution

## Abstract

Cooking with kerosene emits toxic pollutants that may impact pregnancy outcomes. Sixty-eight women in their first trimester of pregnancy, kerosene users (*n* = 42) and liquefied natural gas (LNG) users (*n* = 26), were followed until birth. Maternal and cord blood were collected immediately after birth. Levels of micronutrients and heavy metals were quantified. Pregnancy outcomes (gestation age (GA), birth weight (BW), and chest and head circumference) were also measured. Mean (± standard deviation (SD)) age of mothers in kerosene and LNG groups were similar (*p* = 0.734). Mean (±SD) BW of newborns of LNG users was significantly higher compared to newborns of kerosene users (3.43 ± 0.32 vs. 3.02 ± 0.43, *p* < 0.001). Mean GA (in weeks) was similar between the two groups (*p* = 0.532). Women in the kerosene group had significantly higher cord blood levels of zinc, lead, mercury, iodine and vitamin B6 and lower levels of folic acid compared to LNG users (*p* < 0.05). Newborns of kerosene users had reduced levels of zinc, lead, mercury, iodine, vitamins B6 and B12, folic acid, and homocysteine compared with LNG users (*p* < 0.05). Also, cooking with kerosene was significantly associated with reduced birth weight after adjusting for potential confounders (β ± standard error (SE) = −0.326 ± 0.155; *p* = 0.040). Smoke from kerosene stove was associated with reduced birth weight and micronutrients imbalance in mothers and newborns.

## 1. Introduction

Kerosene remains one of the most widely used domestic cooking energy in low- and middle-income countries (LMICs) of Africa, Asia, and Latin America [1]. It is estimated that nearly 500 million households globally rely on kerosene or other liquid fuels for cooking and other energy needs [2] since they are perceived to be cleaner alternatives to solid fuels and coal. Nearly 27% of Nigerians use kerosene for their household cooking energy needs [3], exposing them to high concentrations of toxic pollutants that are emitted during cooking. By contrast, gaseous fuels (liquefied petroleum gas (LPG) and liquefied natural gas (LNG)) that burn with higher combustion efficiency are used in most high-income countries [1]. Kerosene contains numerous health-damaging compounds [1,4]. Levels of cadmium (Cd), zinc (Zn), nickel (Ni), cobalt (Co), copper (Cu) and lead (Pb) were higher in indoor air of Indian households using kerosene, cow dung cake, coal, and wood compared with families using LPG [5]. An emission study from Thai kerosene stove users identified 11 genotoxic polycyclic aromatic hydrocarbons (PAHs), which were higher in kerosene users compared with both wood and sawdust briquette users [6]. Sulfur dioxide and carbon monoxide (CO) levels during cooking with kerosene were found to be twice as high compared with LPG users [7,8]. Exposure to products of kerosene combustion impairs lung function and increases the likelihood of infections including tuberculosis, asthma, and lung cancer due to kerosene-induced inflammation and oxidative stress [1,9]. Nadeau et al. [10] reported that PAHs present in kerosene fuels mediate immunosuppression. The use of LNG for cooking, relative to solid fuels and kerosene, has the least harmful effect on health, although prolonged and occupational exposure to LNG may alter some hematological and biochemical parameters [11].

Exposure to high concentrations of heavy metals such as lead and cadmium during pregnancy increases the likelihood of miscarriages, premature deliveries, low birth weight (LBW), and impaired cognition in growing children [12]. Although the mechanism by which the constituents of household air pollution (HAP) mediates harmful effects is not fully understood, previous studies suggest induction of oxidative stress and inflammation [13] due to interaction between fine particulate matter (PM), PAH, redox-active transition metals, and redox-cycling quinoids [14,15], which, in turn, may affect DNA methylation that plays an important role in developmental and disease pathways.

We investigated the relationships between HAP from kerosene and pregnancy outcomes as well as micronutrient levels in cord blood of mothers and newborns. We hypothesized that exposure to HAP from kerosene will negatively affect pregnancy outcomes as well as micronutrient levels in mothers and newborns.

## 2. Materials and Methods

### 2.1. Study Design, Study Site, and Recruitment of Subjects

**Figure 1:**
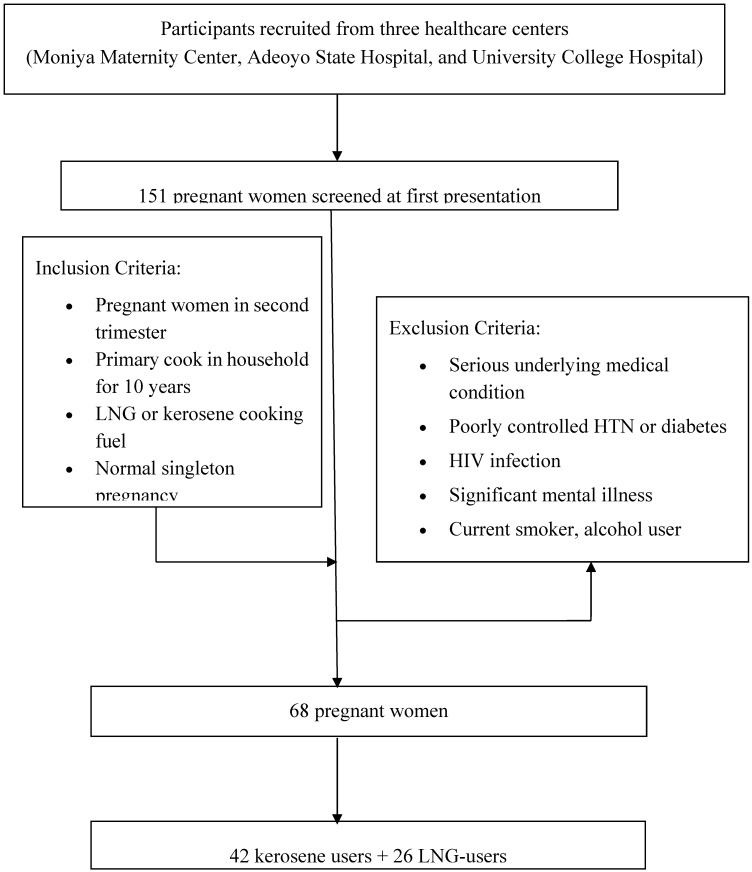
Flow diagram for participant recruitment. HIV: Human immunodeficiency virus; HTN: hypertension; LNG: liquefied natural gas.

This is a cross-sectional study where 68 pregnant women (30.2 ± 5.6 years of age) were recruited from three health centers (Moniya Maternity Centre, a primary health center; Adeoyo State Hospital, a secondary health center and University College Hospital, a tertiary health center) in Ibadan, Nigeria between May and December 2015 (Figure 1). The Ethics Review Board for Oyo State Ministry of Health approved the study protocol (Approval Number: AD 13/479).

Questionnaires were administered at study entry to obtain socio-economic and demographic information. One hundred and fifty-one consecutive pregnant women who cooked with either kerosene, LNG, wood, electricity or a combination of these cooking fuel for the past 10 years were recruited at first presentation to the health center (during first trimester of pregnancy). Among these women, 42 cooked primarily with kerosene; 26 cooked with LNG; 3 cooked with electricity; 3 cooked with firewood; 30 cooked with a combination of kerosene and LNG; 14 cooked with a combination of electricity and LNG; 11 cooked with combination of kerosene, electricity, and LNG; and 5 cooked with combination of kerosene and electricity. Forty-two women who cooked, primarily, with kerosene and 26 who cooked, primarily, with LNG for the past 10 years were considered for this study. The LNG group was used as the reference group for comparability of outcomes. As part of the socio-economic and demographic information, additional variables obtained at study entry included dietary patterns that could impair micronutrient status and nutritional supplements. Exclusion criteria for participation in the study included high-risk pregnancy, poorly controlled hypertension, diabetes mellitus and human immunodeficiency virus (HIV) infection.

All newborns were weighed at birth with an electronic digital scale. The gestational ages of all live births (in weeks) were also estimated within hours of delivery as well as head circumference, crown-to-heel length, chest circumference and placenta weight.

### 2.2. Blood Sample Collection and Analysis

Venous blood (10 mL) samples were obtained from the ante-cubital vein of the participating mothers immediately after delivery. Blood samples from the newborns’ umbilical cords were also obtained at birth into vacutainer tubes that did not contain anticoagulant. Samples were allowed to clot, retract, and were spun at 4000 rpm for 5 min using the MSE Table Centrifuge (MSE, East Sussex, UK). The sera were collected and stored in sterile cryoprecipitate tubes at −20 °C until analysis was performed.

Analyses for zinc, lead and mercury were performed using atomic absorption spectrophotometry. For the analyses of trace metals, serum and standards were appropriately diluted with deionized water. The serum was deproteinized, spun, and supernatant was obtained. This was aspirated into an atomic absorption spectrophotometer (AAS) (GBC Scientific Equipment, Hampshire, IL, USA) for the analysis of trace metals based on the principle that when atoms in the aspirated vapor are excited, they return to ground state by emitting light of the same wavelength. The amount of light absorbed by the metal is proportional to its concentration, which is determined by specific wavelength in the AAS [16].

Vitamins B6 and B12, folic acid, and homocysteine levels were determined using enzyme-linked immunosorbent assay (ELISA) method as previously described [17]. The ELISA kits for vitamins B6 and B12, folic acid, and homocysteine (Alpha Diagnostic International, San Antonio, TX, USA) were used for the analysis. A fixed volume per well of appropriate sample dilution buffer, antigen standard cocktail, or an experimental sample was pipetted into microtiter plates. This sample was incubated at room temperature (25–27 °C) for a specified length of time based on the micronutrient being analyzed. The ELISA immunoplate was washed three times with 350 μL/well of washing buffer. Then 100 μL per well of detection antibodies was added. This mixture was incubated at room temperature for 60 min. The immunoplate was rewashed three times with 350 μL/well of washing buffer. A concentration of 100 μL/well of diluted avidin–horseradish peroxidase (HRP) conjugate was added, after which the plate was incubated at room temperature for 30 min in darkness. The plate was washed four times and 100 μL/well of developing solution was added. The reaction was stopped with 100 μL/well of Stop Solution (Alpha Diagnostic International, San Antonio, TX, USA) and the optical density (OD) was read at specified wavelength within 30 min following the addition of stop solution. The average absorbance value of each OD was plotted against corresponding cytokine values to create a standard curve. The average absorbance of each serum sample was used to determine corresponding cytokine values by interpolating from the curve. The samples were run in duplicates and with the technician being unaware of which fuel group the samples came from.

All field and laboratory personnel were blinded to the exposure status of the women to minimize bias in the measurements of pregnancy outcomes and levels of maternal and fetal cord blood micronutrients.

### 2.3. Data Analysis

Data analysis was performed using SPSS version 17 Statistical package (SPSS Inc., Chicago, IL, USA). Results were expressed as mean ± standard deviation (SD). Mean serum levels of the micronutrients and vitamins were compared using Student’s *t*-test. Pearson’s correlation coefficients (*r*) were used to determine correlations between parameters in maternal and cord blood sera with pregnancy outcome measures like birth weight (BW), placenta weight (PW), crown-to-heel length (C2H), head circumference (HC), chest circumference (CC), and gestational age (GA). Linear regression analysis was performed to assess the relationship between micronutrients and heavy metals and birth weight. Multivariate linear regression analysis was used to examine association between HAP from kerosene and birth weight while adjusting for potential confounders. The variables were included in the final model based on statistical significance, clinical/biological importance, and potential confounding. The Hosmer–Lemeshow test was used to assess model goodness-of-fit. The strength of the associations were assessed using *β* coefficients with standard error. Statistical significance was assigned at *p* < 0.05.

## 3. Results

### 3.1. Socio-Demographic Characteristics

Table 1 shows the socio-demographic characteristics of the participants. The kerosene and LNG users differed significantly in their education status, number of children and annual family income (*p* < 0.05).

### 3.2. Pregnancy Outcomes

Gestational age (GA), head circumference, chest circumference, chest-to-head length of newborns was similar and not statistically different between kerosene and LNG users (Table 2). Placenta weight was higher in newborns of kerosene users compared to LNG users but the difference was not significant (*p* = 0.082).

The mean birth weight of newborns of kerosene-using mothers was significantly lower compared to the birth weight of newborns of LNG-using mothers (*p* < 0.001; Table 2). On average, newborns of kerosene users were 0.41 kg lighter compared to newborns of LNG users. No significant difference was observed for other pregnancy outcomes between the two groups.

### 3.3. Levels of Micronutrients and Heavy Metals

Maternal and cord blood levels of micronutrients (zinc, iodine, vitamin B6, vitamin B12, folic acid, homocysteine) and heavy metals (lead and mercury) are presented in Table 3. Pregnant kerosene users had significantly higher levels of zinc, lead, mercury, iodine and vitamin B6 but reduced levels of folic acid compared with LNG users. Cord blood from newborns of kerosene users exhibited significantly reduced levels of zinc, lead, mercury, iodine, vitamins B6 and B12, folic acid, and homocysteine compared with newborns from LNG users.

We also examined associations between maternal blood micronutrients and heavy metals levels and birth weight in the two cooking fuel groups (Table 4). Overall, lead, mercury, and iodine levels were significantly associated with reduced birth weight. However, after stratification by fuel groups, the reduced birth weight associated with maternal lead, mercury, and iodine levels was still evident in both groups but no longer significant except for the maternal blood lead level which was significantly associated with reduced birth weight in the newborns of kerosene users. Other micronutrients and heavy metals showed no consistent associations with birth weight.

### 3.4. Correlation between Maternal and Fetal Micronutrients Levels

Table 5 shows the correlation of micronutrient status between mothers who cooked with either kerosene or LNG and cord sera of their newborn babies. Statistically significant negative correlations were observed for maternal and cord zinc, iodine, vitamin B6, vitamin B12, folic acid and homocysteine levels.

To confirm that the observed significant difference in birth weight between groups was not explained by socio-economic characteristics that were statistically significant between fuel groups (family income, education level, number of children) and differences in the levels of maternal micronutrients and heavy metals (zinc, lead, mercury, iodine, B6, and folic acid), we performed multiple linear regression analysis adjusting for these variables in addition to mother’s age and multivitamin consumption during pregnancy. The association remained consistent with newborns of kerosene-using mothers having significantly reduced birth weight (*β* ± standard error (SE) = −0.326 ± 0.155; *p* = 0.040). The reduced birth weight of newborns was also evident when micronutrients and heavy metals in cord blood were included in a separate model adjusting for socio-economic characteristics, mother’s age, and multivitamins consumption during pregnancy (*β* ± SE = −0.413 ± 0.192; *p* = 0.036).

## 4. Discussion

This study investigated pregnancy outcomes and levels of micronutrients and heavy metals in pregnant women cooking with kerosene and LNG. We observed that kerosene users tend to have newborns with decreased birth weight compared with LNG users suggesting that HAP from kerosene may predict lower birth weight in newborns. While folic acid level was significantly higher among LNG users, levels of zinc, iodine and vitamin B6 were significantly reduced in them compared with kerosene-users. In the cord sera, levels of all essential micronutrients were found to be significantly higher in the LNG group compared with the kerosene group.

In a study from Pakistan, newborns of firewood-using cooking mothers were on average 82 g lighter than newborns of natural gas-using mothers [18]. In another study from Palestine, infants of firewood-using mothers were 309 g lower in birth weight compared to unexposed mothers [19]. In a recently conducted systematic review and meta-analysis to evaluate the strength of evidence on HAP and adverse pregnancy outcomes, Amegah et al. [20] reported that 19 eligible studies showed a combined effect of 86.43 g reduction in birth weight, effect summary (ES) of 1.35 (95% confidence interval (CI): 1.23, 1.48) and 1.29 (95% CI: 1.18, 1.41) and increased risk of low birth weight and stillbirth, respectively. To minimize or eliminate the effects of HAP from biomass fuel, it is suggested that cooking with kerosene is a better alternative compared to biomass fuel. However, we have shown in this study that such an alternative source of domestic cooking energy may not be the best way to prevent adverse pregnancy outcomes, especially in women from LMICs. We demonstrated that prenatal exposure to HAP from cooking with kerosene might also decrease birth weight and contribute to micronutrient imbalance in exposed mothers and infants. Therefore, access to cleaner energy sources listed in the topmost layer of the World Health Organization (WHO) energy ladder (e.g., electricity, LNG, and ethanol) [21] could be the safest options to combat and mitigate the adverse effects of HAP in women in LMICs. Cooking with kerosene leads to emission of many pollutants that have adverse pregnancy outcomes like stillbirth [22], low birth weight (LBW), and neonatal deaths [23] when compared with LPG or biogas. There are few studies that have rigorously investigated the influence of HAP from kerosene use for cooking on pregnant women in a randomized controlled trial [9,24,25].

Diet is a key factor in determining genomic stability, as it affects all relevant developmental pathways (i.e., activation/detoxification of chemicals preventing DNA oxidation, DNA repair, apoptosis and DNA synthesis) [26]. Zinc, folic acid, and vitamins are essential micronutrients that play important roles in DNA transcription and methylation. One-carbon metabolism, an essential metabolic process that ultimately provides the methyl group for DNA methylation reactions, requires adequate intake of methyl-donor nutrients such as folate and methylation cofactors including vitamin B12 and zinc [27], but some of these micronutrients, for instance, zinc are antagonized by heavy metals such as lead and mercury [28]. Folate and vitamin B12 deficiencies cause DNA hypo methylation, a defect that leads to chromosome loss [29,30]. Folate deficiency among women of childbearing age is also a risk factor for LBW and preterm delivery [31]. Vitamin B12 is important in synthesis of methionine, an essential component in methylation process and vitamin B6 is essential in generation of folate [30]. Since the fetus is totally dependent on the mother for the supply of nutrients essential to maintain viability, optimal maternal nutrition reduces the risks of preterm delivery and LBW [32], improves postnatal growth, and reduces maternal and infant morbidity and mortality [33,34], thus inverse relations between maternal and cord levels of micronutrients. Vitamins A, C, and E are essential during pregnancy, as they promote general growth, ameliorate oxidative stress, and provide antioxidant defense to the fetus [35]. Folate plays a significant role as a coenzyme in the synthesis of DNA, RNA, and certain amino acids and in supporting normal fetal development [36]. Zinc is an integral part of many enzymes in the body with an important role in nucleic acid metabolism, cell replication, tissue repair, and growth [37]. It is also an essential co-factor for the enzyme DNA methyltransferases, which is involved in the methylation process [38]. The increased levels of zinc, iodine and vitamin B6 observed among kerosene users might represent compensation for low levels of folic acid and vitamin B12, which may suggest impaired DNA methylation. Both folate and vitamin B12 also play roles in metabolic pathways that maintain adequate homocysteine levels [39]. They are needed in the re-methylation of homocysteine to methionine. An increased homocysteine level has been associated with atherogenesis and it is a risk factor for coronary artery disease [40], pregnancy loss [41] and neural tube defects [42]. These indicate that adequate levels of essential nutrients in the diet are important to maintain optimal level of homocysteine. Increased homocysteine levels in pregnant women exposed to kerosene smoke compared with LNG users might suggest that women cooking with kerosene may be predisposed to increased risk of adverse pregnancy outcomes. Although the neural tube defect was not observed in the present study, increased serum homocysteine in our pregnant women using kerosene may portend danger for the mother and developing infant.

Lead and mercury are cumulative toxicants that are particularly harmful to young children. Childhood lead exposure is estimated to contribute to about 600,000 new cases of intellectual disabilities in children every year and no known level of lead exposure is considered safe [43]. We posit that ingestion of heavy metals such as lead and cadmium by mothers during pregnancy may increase the possibility of miscarriages, LBW, and impaired cognitive function in growing young children [12]. In this study, increased cord blood levels of lead and mercury were observed in the LNG group, which might be from impurities in fuel and from environmental exposures. Our observation is consistent with previous studies, which associated LPG use with increased respiratory risk [21,44,45,46,47] and that gas stoves produce NOx which should only be used with adequate ventilation particularly a hood, as is recommended in most developed countries [48]. The observation that birth weight, chest circumference, and gestational age were negatively correlated with cord blood levels of mercury, lead, iodine, vitamin B6, and homocysteine in babies of kerosene users suggests that cooking with kerosene by pregnant women resulted in more adverse pregnancy outcomes and that these may be due to low levels of methylation-essential micronutrients (iodine, vitamin B6 and homocysteine) during pregnancy.

The limitation of the study is the low sample size. Due to some cultural beliefs and religion preferences, it was difficult to convince participants to deliver their babies at health care facilities. However, since we observed statistical significance in pregnancy outcomes and many of the micronutrients, we feel that the sample size was adequate to assess our hypothesized effects. In Nigeria, there is no free or subsidized healthcare policy, which may suggest that only participants with moderate to high socio-economic status participated in the study. Therefore, the results of the study may be generalized to pregnant women attending healthcare centers during pregnancy in the study area. We suggest that caution be observed when comparing the results of this study to other studies that might have recruited pregnant women from both government-established healthcare centers and local traditional delivery centers. The two fuel groups in this study were distinct. LNG users were women that used primarily LNG for cooking. Similarly, kerosene users were women that used primarily kerosene for cooking. While we ensured the two fuel groups used kerosene and LNG predominantly, the women might have used other additional cooking methods or were exposed to other cooking fuel types during the study period due to communal lifestyles that are typical of women in this setting. Finally, we did not determine whether the women in this study cooked by themselves and how long they stayed in the kitchen cooking during the follow-up period. This would have enabled us to determine the degree of exposure during the pregnancy period. However, with the cultural etiquette in Nigeria, and similar African countries, where women are responsible for domestic cooking, we are confident that the majority of women in this study participated in cooking during the follow-up period. Also, our previous study in the settings where the current study was performed showed that 61% of women cooked approximately 3–5 h, on average, per day [49].

The strength of the study is the objective measures used in all outcomes assessed which limits or greatly minimized the misclassification of outcomes. Also, we limited participants to pregnant women using kerosene or LNG predominantly for 10 years. While this might have reduced the number of participants and limited our sample size, it helped to minimize exposure misclassification and cross effects of firewood smoke exposure, which has been reported to be associated with adverse pregnancy outcomes [20].

## 5. Conclusions

The study showed that pregnant women who cook with kerosene experienced more adverse pregnancy outcomes with their infants weighing less compared to mothers using LNG for cooking. We also observed micronutrient imbalance in mothers and newborns exposed to HAP from kerosene. The results of the study underscore the need to improve nutrition during pregnancy and to strengthen and formulate policies to replace kerosene as cooking and household fuel with much cleaner fuels like LNG and ethanol.

## Figures and Tables

**Table 1:** Characteristics of study population by cooking fuel group.

Characteristics	Kerosene Users (*N* = 42)	LNG Users (*N* = 26)	*p*-Value
Age, years (mean (SD))	29.48 (4.54)	28.42 (5.33)	0.391
Parity (mean (SD))	3.24 (1.74)	2.50 (2.14)	0.123
Multivitamins use during pregnancy (%)	4.8	3.8	0.862
Daily vegetable consumption during pregnancy (%)	66.7	61.5	0.674
Passive cigarette smokers (%)	4.8	19.2	0.056
Family income per year			
Low (<N250,000) (%)	78.6	30.8	<0.001 *
Medium (≥N250,000 and <N500,000) (%)	14.3	3.8	-
High (≥N500,000) (%)	7.1	65.4	-
Education above high school (%)	14.3	46.2	0.001 *
Number of children			
1–2 (%)	11.9	52.0	<0.001 *
3–4 (%)	45.2	40.0	-
>4 (%)	42.9	8.0	-
Married (%)	61.9	65.4	0.768
Modern homes (%)	52.1	45.9	0.153

* *p* < 0.05; LNG: Liquefied natural gas; SD: Standard deviation.

**Table 2:** Pregnancy outcomes of mothers by cooking fuel group.

Pregnancy Outcomes	Kerosene Users (*N* = 42)	LNG Users (*N* = 26)	*p*-Value
Gestational Age, weeks (mean (SD))	35.46 (3.05)	34.98 (3.12)	0.532
Birth weight, kg (mean (SD))	3.02 (0.43)	3.43 (0.32)	<0.001
Head circumference, cm (mean (SD))	35.14 (3.20)	35.54 (3.89)	0.655
Chest circumference, cm (mean (SD))	35.26 (3.58)	34.46 (3.14)	0.354
CTH length, cm (mean (SD))	46.43 (3.89)	45.23 (4.02)	0.232
Placenta weight, kg (mean (SD))	0.82 (0.09)	0.77 (0.13)	0.082

BW: Birth weight; CTH: Crown-to-heel length; GA: Gestational age; SD: Standard deviation.

**Table 3:** Mean (±SD) micronutrient levels in maternal and cord blood cooking fuel group.

Heavy Metals and Micronutrients	Kerosene Users (*N* = 42)	LNG Users (*N* = 26)	*p*-Value
**Maternal blood**			
Zinc (Zn)	95.43 (16.06)	77.14 (16.26)	<0.001 *
Lead (Pb)	5.23 (0.94)	4.23 (0.99)	<0.001 *
Mercury (Hg)	9.88 (1.62)	7.98 (0.93)	<0.001 *
Iodine (I)	57.50 (12.02)	48.85 (5.70)	<0.001 *
B6	8.71 (1.45)	6.74 (0.93)	<0.001 *
B12	436.41 (86.64)	461.74 (90.65)	0.254
Folic acid	6.68 (1.70)	7.59 (1.54)	0.026 *
Homocysteine	7.72 (1.17)	6.68 (1.70)	0.111
**Fetal cord blood**			
Zinc (Zn)	92.78 (19.14)	124.28 (24.90)	<0.001 *
Lead (Pb)	4.45 (1.95)	5.77 (2.25)	0.017
Mercury (Hg)	8.32 (3.50)	10.72 (4.00)	0.015
Iodine (I)	49.62 (20.80)	65.60 (19.27)	0.002 *
B6	7.31 (2.13)	11.23 (3.04)	<0.001 *
B12	249.95 (82.54)	346.26 (49.90)	<0.001 *
Folic acid	7.72 (2.04)	10.93 (1.57)	<0.001 *
Homocysteine	7.75 (1.36)	10.42 (1.36)	<0.001 *

* *p* < 0.05; SD: Standard deviation.

**Table 4:** Associations^†^ between micronutrients and heavy metals in maternal blood and birth weight stratified by cooking fuel groups.

Heavy Metals and Micronutrients	Overall (*N* = 67)	Kerosene Users (*N* = 42)	LNG Users (*N* = 26)
	*β* (SE)	*β* (SE)	*β* (SE)
Zinc (Zn)	−0.002 (0.003)	0.004 (0.004)	0.003 (0.004)
Lead (Pb)	−0.159 (0.047) *	−0.030 (0.072) *	−0.185 (0.055)
Mercury (Hg)	−0.098 (0.030) *	−0.034 (0.042)	−0.102 (0.068)
Iodine (I)	−0.014 (0.005) *	−0.007 (0.006)	−0.017 (0.011)
B6	−0.064 (0.033)	0.016 (0.047)	0.033 (0.071)
B12	0.001 (0.001)	0.001 (0.001)	0.000 (0.001)
Folic acid	0.012 (0.032)	−0.015 (0.040)	−0.035 (0.042)
Homocysteine	−0.014 (0.029)	0.009 (0.032)	−0.003 (0.056)

^†^ Model adjusted for family income, maternal age, and education level. * *p* < 0.05; LNG, liquefied natural gas. SE: Standard error.

**Table 5:** Correlations between maternal and fetal micronutrient levels.

Mother/Fetal Cord	Zinc	Iodine	B6	B12	Folic Acid	Homocysteine
Zinc	−0.48 **	−0.37 **	−0.48 **	−0.35 **	−0.52 **	−0.51 **
Iodine	-	0.02	−0.32 **	−0.29 **	−0.24 **	−0.25 *
B6	-	-	−0.56 **	−0.30 *	−0.37	−0.56 **
B12	-	-	-	0.21	0.16	0.04
Folic acid	-	-	-	-	0.15	0.14
Homocysteine	-	-	-	-	-	−0.09

* *p* < 0.05; ** *p* < 0.001.
